# Structural mechanism of AadA, a dual-specificity aminoglycoside adenylyltransferase from *Salmonella enterica*

**DOI:** 10.1074/jbc.RA118.003989

**Published:** 2018-06-05

**Authors:** Ana Laura Stern, Sander Egbert Van der Verren, Sandesh Kanchugal P, Joakim Näsvall, Hugo Gutiérrez-de-Terán, Maria Selmer

**Affiliations:** From the ‡ Department of Cell and Molecular Biology, Uppsala University, BMC, Box 596, SE-751 24 Uppsala, Sweden and; § Department of Medical Biochemistry and Microbiology, Uppsala University, BMC, Box 582, SE-751 23 Uppsala, Sweden

**Keywords:** antibiotic resistance, enzyme structure, enzyme mechanism, X-ray crystallography, antibiotics, ATP, spectinomycin, streptomycin

## Abstract

Streptomycin and spectinomycin are antibiotics that bind to the bacterial ribosome and perturb protein synthesis. The clinically most prevalent bacterial resistance mechanism is their chemical modification by aminoglycoside-modifying enzymes such as aminoglycoside nucleotidyltransferases (ANTs). AadA from *Salmonella enterica* is an aminoglycoside (3″)(9) adenylyltransferase that *O-*adenylates position 3″ of streptomycin and position 9 of spectinomycin. We previously reported the apo-AadA structure with a closed active site. To clarify how AadA binds ATP and its two chemically distinct drug substrates, we here report crystal structures of WT AadA complexed with ATP, magnesium, and streptomycin and of an active-site mutant, E87Q, complexed with ATP and streptomycin or the closely related dihydrostreptomycin. These structures revealed that ATP binding induces a conformational change that positions the two domains for drug binding at the interdomain cleft and disclosed the interactions between both domains and the three rings of streptomycin. Spectinomycin docking followed by molecular dynamics simulations suggested that, despite the limited structural similarities with streptomycin, spectinomycin makes similar interactions around the modification site and, in agreement with mutational data, forms critical interactions with fewer residues. Using structure-guided sequence analyses of ANT(3″)(9) enzymes acting on both substrates and ANT(9) enzymes active only on spectinomycin, we identified sequence determinants for activity on each substrate. We experimentally confirmed that Trp-173 and Asp-178 are essential only for streptomycin resistance. Activity assays indicated that Glu-87 is the catalytic base in AadA and that the nonadenylating E87Q mutant can hydrolyze ATP in the presence of streptomycin.

## Introduction

Streptomycin and spectinomycin are antibiotics that bind to the bacterial ribosome and interfere with protein synthesis through effects on decoding and translocation (for reviews, see Refs. 1–3). They belong to the aminoglycoside and aminocyclitol families of antibiotics, both carrying amino groups that make the drugs positively charged, allowing their specific binding to negatively charged binding sites in 16S rRNA (4, 5). Streptomycin and spectinomycin are both in clinical use (6) and included in the World Health Organization model list of essential medicines. The closely related derivative dihydrostreptomycin (dhs)^4^ is only used in veterinary medicine due to its ototoxicity (7).

Clinically, the most important resistance mechanism to these drugs is their inactivation by aminoglycoside-modifying enzymes, but resistance can also occur by mutations in the drug target or through mechanisms of decreased uptake or increased efflux (3, 8). Modification of streptomycin and spectinomycin by *O*-phosphorylation or *O*-nucleotidylation prevents the drugs from binding to their respective binding sites on the ribosome (for reviews, see Refs. 9 and 10).

**Figure 1.**
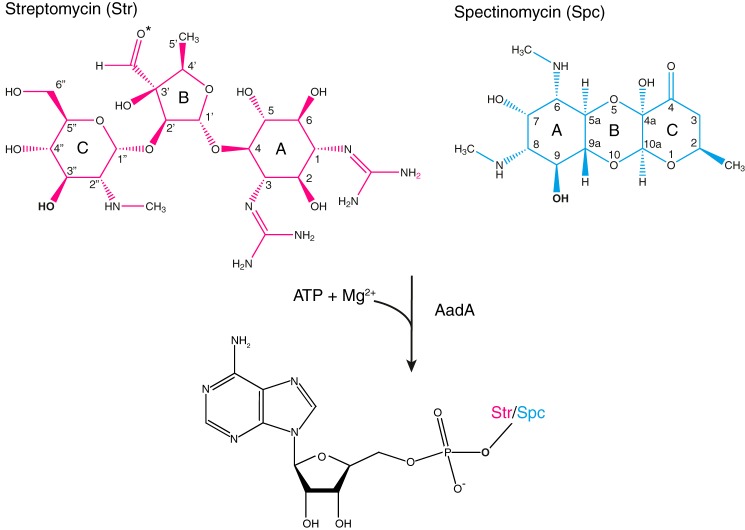
**Chemical reaction catalyzed by AadA.** The sites of *O*-adenylation on streptomycin (*Str*) and spectinomycin (*Spc*) are shown in *bold*. The carbonyl group that is reduced in dihydrostreptomycin is marked with an *asterisk*.

Aminoglycoside (3″)(9) adenylyltransferase AadA from *Salmonella enterica* belongs to the ANT(3″)-Ia family (9) and catalyzes the magnesium-dependent *O*-adenylation of streptomycin and spectinomycin at positions 3″ and 9, respectively (11) (Fig. 1). The two drugs are chemically dissimilar with streptomycin containing three *O*-linked rings with significant conformational freedom, whereas spectinomycin is a conformationally more restrained tricyclic molecule.

We previously determined the apo crystal structure of *S. enterica* AadA at 2.5-Å resolution (12). In the apo state, AadA has a monomeric two-domain structure with the active site located between an N-terminal adenylyltransferase domain and a C-terminal helical domain. In this inactive conformation, residues predicted to be involved in binding of ATP and magnesium were instead engaged in interdomain interactions. Using isothermal titration calorimetry (ITC), we demonstrated that magnesium and ATP had to bind prior to aminoglycoside substrate, suggesting that ATP binding triggered a conformational change, repositioning the two domains for substrate recognition.

We now set out to characterize how AadA binds to ATP and its two different antibiotic substrates. We here present crystal structures of wild type (WT) AadA with ATP or ATP and streptomycin and of an active-site mutant in complex with ATP and streptomycin or dihydrostreptomycin. Using manual docking and molecular dynamics (MD) simulations, we present a model for how spectinomycin explores a partially different part of the active-site pocket. We also confirm the identity of the catalytic base using *in vitro* assays. Based on structure-based sequence analysis, we propose and experimentally verify sequence determinants that allow classification of ANT(3″)(9) enzymes and the related ANT(9) enzymes, which only mediate resistance to spectinomycin.

## Results and discussion

### Binding of ATP and magnesium repositions the two domains of AadA

Cocrystallization of WT AadA with ATP and magnesium yielded well-diffracting crystals. The crystals grew in a new space group, P3_2_, with two molecules in the asymmetric unit, and the complete atomic structure could be built and refined to 1.9-Å resolution. The two molecules of AadA display close-to-identical structures (root mean square deviation (r.m.s.d.) of 0.1 Å over 261 C^α^ atoms). This structure represents a native state of the enzyme prior to binding of aminoglycoside substrate.

**Figure 2.**
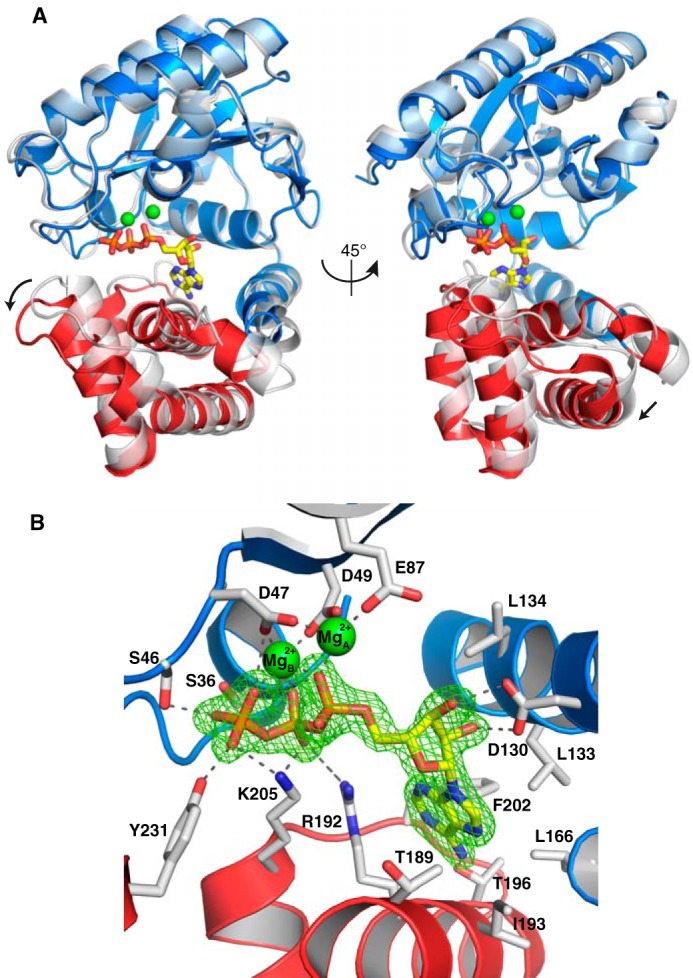
**Structure of AadA in complex with ATP and magnesium.** *A*, binding of ATP (*yellow*) and two magnesium ions (*green*) triggers a shift compared with the apo structure (*transparent gray*), indicated by an *arrow*, of the main part of the C-terminal domain (*red*) relative to the N-terminal domain and helix 7 (*blue*). *B*, detailed interactions of AadA with ATP (*yellow*) and magnesium ions (*green*). Direct hydrogen bonds between protein and ligands are shown as *dashed lines*, and the *F_o_* − *F_c_* omit map for ATP, contoured at 5 σ, is shown as *green mesh*.

To accommodate binding of ATP and magnesium, compared with the apo structure, the main part of the C-terminal domain rotates 11° relative to the N-terminal domain around the first helix of the C-terminal domain (helix 7), resulting in a shift of up to 4 Å (Fig. 2*A*). There are also local conformational changes of the 202–206 loop region directly linked to the interaction with ATP. The two parts of the structure are very similar to the apo structure (12) (r.m.s.d. of 0.77 Å over 171 C^α^ atoms for residues 1–173 and 1.03 Å over 88 C^α^ atoms for residues 175–262).

The structure shows clear electron density for ATP and two magnesium ions in the interdomain cleft (Fig. 2*B*). In the N-terminal domain, Asp-47, Asp-49, and Glu-87 together with the phosphates of ATP coordinate the two magnesium ions. In addition, the phosphates make interactions with Ser-36 and Ser-46 in the N-terminal domain and Arg-192, Lys-205, and Tyr-231 in the C-terminal domain. The ribose is in C2′ *endo* conformation with both hydroxyl groups involved in hydrogen bonding to Asp-130. The adenine base is in *syn* conformation, packing between Leu-133, Leu-166, Thr-189, Arg-192, Ile-193, and Phe-202, and makes a single hydrogen bond to Thr-196 of the protein.

The interaction of Arg-192 with the bridging oxygen seems critical to obtain a magnesium-coordinating conformation of ATP because binding of nonhydrolyzable ATP analogues AMPCPP (12) and AMPNPP (data not shown) could not be detected. In the apo crystal structure, ATP- and magnesium-binding residues of both domains were involved in interdomain interactions, explaining why that crystal form could not accommodate any ligand binding (12).

The magnesium- and phosphate-coordinating residues in the N-terminal domain are to a large extent conserved in other adenylyltransferase enzymes as well as polymerases and nucleases with two metal ions in the active site (12, 13) (see below). We can thus rationalize that some related structures, *e.g.* the structure of kanamycin nucleotidyltransferase in complex with AMPCPP (14), only display one magnesium ion because the bridging carbon of the ATP analogue affects the conformation of the phosphate tail in a manner preventing binding of a second magnesium ion.

### Structures of AadA in complex with streptomycin and dihydrostreptomycin

Previous experiments demonstrated that the E87Q mutant of AadA did not convey resistance to streptomycin but still bound ATP and streptomycin, although with 4- and 20-fold lower affinity than the WT enzyme (12). We now cocrystallized this presumably catalytically inactive mutant with ATP, magnesium, and antibiotic substrates and obtained well diffracting crystals with streptomycin and dihydrostreptomycin. The crystals grew in the same space group and with similar cell dimensions as the WT crystals with ATP but only appeared in conditions containing calcium. The structure of AadA(E87Q) with ATP and streptomycin (AadA(E87Q)–ATP–sry) was refined to 1.73-Å resolution, and the structure of AadA(E87Q) with ATP and dihydrostreptomycin (AadA(E87Q)–ATP–dhs) was resolved to 1.4-Å resolution. Both structures are modeled with calcium ions instead of magnesium in the active site. The identity of divalent ion B was confirmed in an anomalous difference map (Fig. S1). The second ion (A) is tentatively modeled as calcium based on coordination distances and level of electron density. However, the identity of this ion does not affect any of our conclusions. Dihydrostreptomycin only differs from streptomycin in the reduction of the carbonyl group at the 3′-position (Fig. 1), and because this position does not show any interaction with the protein and the two structures are virtually identical (r.m.s.d. of 0.2 Å over 261 C^α^ atoms), further analysis will be based on the dihydrostreptomycin structure.

**Figure 3.**
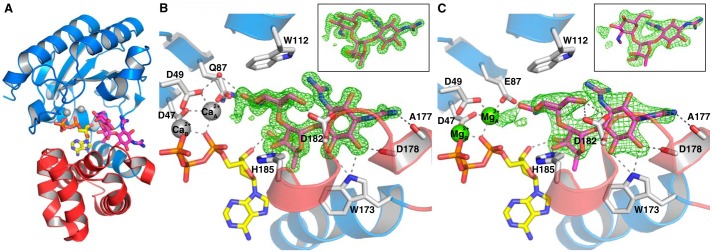
**Structures of AadA in complex with aminoglycosides and ATP.** *A*, dihydrostreptomycin (*magenta*) binds to the interdomain cleft, next to ATP (*yellow*), in AadA(E87Q). The orientation is identical to the second view of Fig. 2*A. B*, detailed interactions of dihydrostreptomycin (*magenta*) in the AadA(E87Q)–ATP–dhs structure with calcium. Direct hydrogen bonds between protein and ligands are shown as *dashed lines*, and the *F_o_* − *F_c_* omit map for dihydrostreptomycin, contoured at 3.5 σ, is shown as *green mesh*. The *inset* shows the ligand and map rotated 30°. *C*, detailed interactions of streptomycin (*magenta*) in the WT AadA–ATP–sry structure with magnesium. The *F_o_* − *F_c_* omit map for streptomycin, contoured at 3.0 σ, is shown as *green mesh*. The *inset* shows the ligand and map rotated 30°.

The overall structure of AadA(E87Q)–ATP–dhs is very similar to AadA–ATP (r.m.s.d. of 0.3 Å over 252 C^α^ atoms). Dihydrostreptomycin is bound between the two domains of AadA with the C ring sandwiched between the two domains in proximity of ATP, whereas the B- and A rings extend further toward the surface and form their major interactions with the C-terminal domain (Fig. 3*A*).

AadA hides 417 Å^2^ (58%) of the van der Waals area of dihydrostreptomycin and makes interactions with all three rings (Fig. 3*B*). The C ring is the site of modification and is situated in proximity of the suggested catalytic base Glu-87 (mutated to glutamine in the structure) with the 3″-hydroxyl situated in the closest position. Furthermore, the ring is also oriented through a hydrogen bond of Asp-182 with the 6″-hydroxyl group and a stacking interaction with Trp-112. In turn, the B ring is oriented by His-185 through a hydrogen bond with the 3′-hydroxyl group. The enzyme does not interact with the carbonyl at position 3′ of the B ring (Fig. 1), showing why AadA can bind and modify both streptomycin and dihydrostreptomycin. Finally, the A ring displays multiple interactions with the enzyme. Hydrogen bond interactions occur between Trp-173 and hydroxyl groups at positions 5 and 6, between the backbone carbonyl of Ala-177 and the guanidinium group at position 1, and between the backbone carbonyl of Asp-178 and the 6-hydroxyl group. The conformational change of the C-terminal domain relative to the N-terminal domain upon ATP binding (Fig. 2*A*) is essential to allow streptomycin to fit into the active site.

Brief soaking of WT AadA–ATP crystals with streptomycin resulted in a 2.05-Å–resolution structure with clear density for ATP, magnesium, and the major part of streptomycin. The overall conformation of the enzyme is very similar to AadA–ATP (r.m.s.d. of 0.3 Å over 256 C^α^ atoms) and AadA(E87Q)–dhs (r.m.s.d. of 0.4 Å over 256 C^α^ atoms). Absence of electron density for part of the C ring (C2″, C3″, and the methylamine; Fig. 3*C*) suggests that this part of the substrate may move within the active site in connection with catalysis (further discussed below).

### Glu-87 as catalytic base of AadA

Sequence analysis and structure comparison with other adenylyltransferase enzymes suggested that Glu-87 is the catalytic base in AadA. This was supported by minimum inhibitory concentration tests of streptomycin with WT AadA and single-amino acid substitution mutants E87Q and E87A (12). To confirm the enzymatic *in vitro* activity of AadA toward streptomycin and to further investigate the role of Glu-87, *in vitro* activity of AadA was tested for the WT and mutant enzymes E87A and E87Q. In the enzymatic assay, pyrophosphatase converted the pyrophosphate by-product of the reaction to phosphate, which was subsequently detected by the malachite green assay.

At the conditions assayed, the turnover for the WT enzyme was 0.020 ± 0.001 s^−1^. To our surprise, both mutant enzymes showed activity in this assay with turnover of 0.032 ± 0.003 s^−1^ for E87Q and 0.004 ± 0.001 s^−1^ for E87A. In the absence of streptomycin, none of the WT or mutant enzymes showed detectable activity.

To distinguish whether the observed activity was a result of streptomycin adenylation or unproductive ATP hydrolysis, we developed a chromatographic assay to directly detect adenylated streptomycin. The positive charge of adenylated streptomycin allows its separation from negatively charged ATP and AMP by cation-exchange chromatography, and detection by absorbance at 260 nm, where unmodified streptomycin does not absorb.

**Figure 4.**
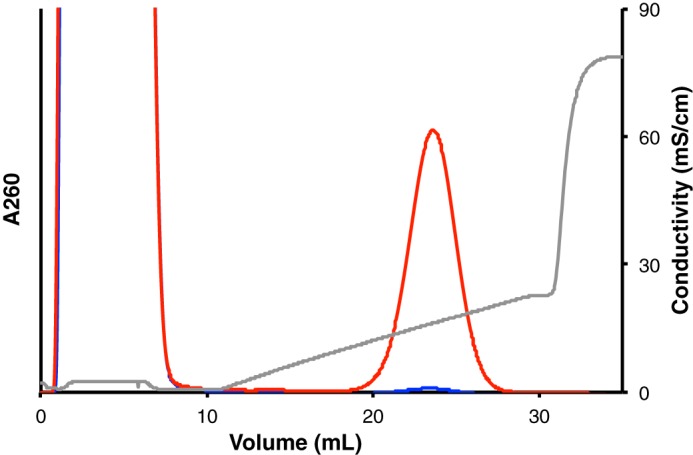
**Ion-exchange chromatogram of adenylation assay with AadA WT (*red*) and E87Q (*blue*).** Adenylated streptomycin elutes at 23.6 ml. *mS*, millisiemens.

Our results showed that, in contrast to the WT enzyme, the E87Q mutant could not adenylate streptomycin (Fig. 4), consolidating the role of Glu-87 as essential for adenylation activity. However, in the absence of catalytic base, water can apparently act as a nucleophile, turning AadA into a hydrolytic enzyme. One such candidate water molecule is in the AadA–ATP–dhs structure coordinated between the magnesium ion, the 3″-hydroxyl of dihydrostreptomycin, and the α-phosphate of ATP (Fig. S2).

We used the H++ server (15) to computationally predict a p*K_a_* of 7.1 for Glu-87 in the ATP structures with magnesium (with or without streptomycin). This indicates that Glu-87 within the catalytic center has a p*K_a_* in the range where it could be reversibly protonated and abstract a proton from the 3″-hydroxyl of streptomycin.

### Structural mechanism of adenylation

The adenyl transfer reaction by AadA is equivalent to the reactions carried out by well characterized two-metal-ion polymerases and nucleases that display very similar arrangements of residues in the active site (13). In analogy with these associative-mechanism enzymes, we predict that the catalytic base Glu-87 will abstract a proton from the 3″-hydroxyl group of streptomycin to allow the oxygen to make a nucleophilic attack on the α-phosphate of ATP. The geometry of the substrates in the active site is consistent with a single-in-line displacement mechanism as suggested for kanamycin nucleotidyltransferase (14) and LinB (16), but the two substrates are too far apart to represent a catalytic state according to this mechanism. Thus, the nucleophilic oxygen would normally coordinate the magnesium ion and approach 2.5-Å distance of the ATP phosphorus atom during the reaction, whereas the position of our 3″-hydroxyl in AadA–ATP–sry is at 5.1 Å from the α-phosphorus. It seems necessary for the substrate to move, possibly linked to a local conformational change of the enzyme active site, to allow the deprotonated 3″-hydroxyl to coordinate the magnesium ion before nucleophilic attack. The weak electron density for the reacting part of the substrate in the AadA–ATP–sry structure supports dynamics in this part of the substrate. In our higher-resolution substrate structures, the active site in the presence of calcium is distorted from the native, magnesium-bound state through a cumulative effect of increased coordination distances. Binding of calcium to the first metal-binding site (B in Figs. 2*B* and 3*B*) increases the distances to coordinating atoms, and the octahedral coordination geometry becomes nonperfect (Fig. S3, *A* and *B*). This has an impact on the second magnesium site (A in Figs. 2*B* and 3*B*), which now has a nonoptimal configuration, leading to partial-occupancy binding of a divalent ion modeled as calcium (Fig. S3, *C* and *D*). In the WT structure, Glu-87 is positioned to coordinate both the magnesium and the substrate 3″-hydroxyl position, using the same side-chain oxygen in the manner observed in other adenylyltransferase structures (16, 17), at 3.7-Å distance to the 3″-hydroxyl.

### Structure-based sequence analysis

According to the Pfam database (18) the two domains of AadA are classified as an NTP_transf_2 domain (PF01909) and a DUF4111 domain (PF13427). Of the 186 proteins classified in Pfam with this architecture, nine have been functionally characterized and can be divided into three classes. The first class of enzymes shows ANT(3″)(9) activity and confers resistance to streptomycin and spectinomycin (AadAs from *S. enterica* (11), *Enterococcus faecalis* (19), *Escherichia coli* (20), *Pseudomonas aeruginosa* (21), and *Serratia marcescens* (22)). The second class of enzymes shows ANT(9) activity and confers resistance to spectinomycin but not to streptomycin (ANT(9)s from *E. faecalis* (19), *Staphylococcus aureus*, and *Campylobacter jejuni* (23)). The third class of enzymes does not provide resistance to any of the antibiotics (one example from *C. jejuni* (23)). Including the functionally annotated sequences that were not in the Pfam database, 193 sequences of presumably the same monomeric domain arrangement and sequence identity of 27% or higher were subjected to subsequent analysis.

**Figure 5.**
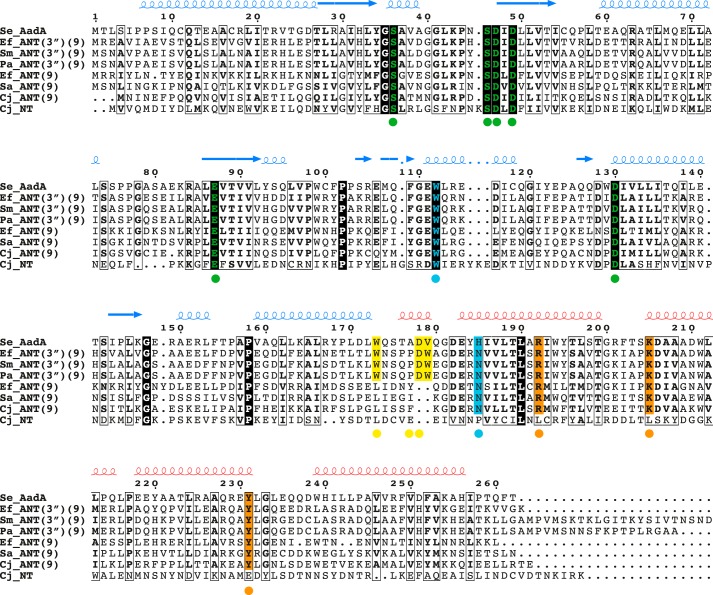
**Multiple sequence alignment of *S. enterica* AadA (Q8ZPX9; UniProt accession numbers in parentheses) with three characterized ANT(3″)(9) enzymes from *E. faecalis* (Q71UU1), *S. marcescens* (Q8VQN7) and *P. aeruginosa* (Q9RGC2); three ANT(9) enzymes from *E. faecalis* (Q07448), *S. aureus* (P0A0D2), and *C. jejuni* (Q4VR99); and one enzyme lacking ANT(3″) and ANT(9) activity from *C. jejuni* (Q4VR96).** Secondary structure of AadA is indicated above the alignment, colored as in Figs. 2 and 3. *Colored dots* indicate contacts with ligands; corresponding sequence conservations are marked in the alignment. *Green*, residues in N-terminal domain coordinating Mg^2+^ and ATP; *orange*, residues in C-terminal domain coordinating the phosphates of ATP; *cyan*, residues coordinating rings B and C of streptomycin; *yellow*, residues coordinating ring A of streptomycin.

To identify the sequence determinants for the ANT(9) and ANT(3″) activities, the substrate-binding residues from the AadA complex structures with streptomycin and dihydrostreptomycin were mapped to the functionally annotated sequences (Fig. 5) and the entire family. The sequence identities between sequences with predicted ANT(3″)(9) activity were 43% or higher, and those between sequences with predicted ANT(9) activity were 38% or higher.

Amino acids that interact with the C ring (the adenylation site) of streptomycin, Glu-87, Trp-112, Asp-182, and His/Asn-185, are conserved in all ANT(3″)(9) and ANT(9) enzymes, defining a subgroup of in total 71 sequences. Enzymes with ANT(3″)(9) activity are characterized by conservation of residues that interact with the A ring of streptomycin. Trp-173 and a two-amino-acid insertion at position 177-178, involved in backbone carbonyl interactions, are conserved in 26 of the 71 sequences. Based on this, we propose that the determinants for adenylation activity on spectinomycin are Glu-87, Trp-112, Asp-182, and His/Asn-185 and that the activity toward streptomycin in addition requires Trp-173 and the insertion at position 177-178. In the characterized enzyme without activity toward spectinomycin or streptomycin (23), only the residues in the N-terminal domain that coordinate ATP and magnesium are conserved, indicating that this and 121 additional enzymes in the Pfam data set with similar architecture may perform a different function.

### Experimental validation of determinants for activity on streptomycin

**Figure 6.**
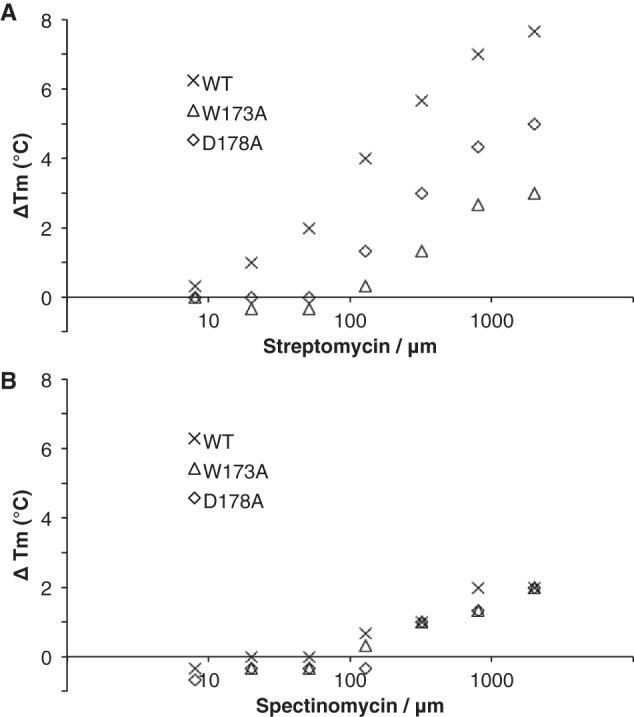
**Thermal stabilization (ΔT*_m_*) of WT and mutant AadA in the presence of streptomycin (*A*) and spectinomycin (*B*).** Δ*T_m_* was calculated as the difference between the average *T_m_* in the presence and absence of ligand.

We hypothesized that the hydrogen bond interaction of Trp-173 with streptomycin and the structure-stabilizing interaction of Asp-178 with the backbone amides of Gln-174 and Ser-175, observed in AadA also in the absence of ATP and streptomycin, would be critical only for resistance to streptomycin. To test this hypothesis, we constructed chromosomal mutants W173A and D178A in the *aadA* gene and subjected the generated strains to *in vivo* minimum inhibitory concentration (MIC) tests with streptomycin and spectinomycin (Table 1). The MIC values for the two mutants were reduced 10- and 5-fold for streptomycin but remained close to WT MIC values for spectinomycin. The same mutations were introduced in AadA for *in vitro* Thermofluor studies. In titrations of WT AadA, streptomycin binding leads to an increase of up to 8 °C in melting temperature (*T_m_*), and spectinomycin binding leads to an increase of 3 °C (Fig. 6). W173A and D178A both destabilize AadA, decreasing *T_m_* from 52 to 50 and 47 °C, respectively. For both mutants, when titrated with streptomycin, temperature stabilization occurs at higher streptomycin concentration compared with WT, indicating weaker binding affinity (Fig. 6*A*). In contrast, the mutants behave as WT when titrated with spectinomycin (Fig. 6*B*). We thus *in vivo* and *in vitro* have experimentally confirmed the sequence determinants for classification of enzymes with ANT(9) or ANT(3″)(9) activity.

**Table 1:** **MICs of streptomycin and spectinomycin for strains with wildtype or mutant AadA**

AadA variant	Strain	MIC (μg ml^−1^)	
		Streptomycin	Spectinomycin
WT	DA6192	128	192
W173A	DA58246	12	192
D178A	DA52256	24	128

### Docking and evaluation of spectinomycin binding to AadA

**Figure 7.**
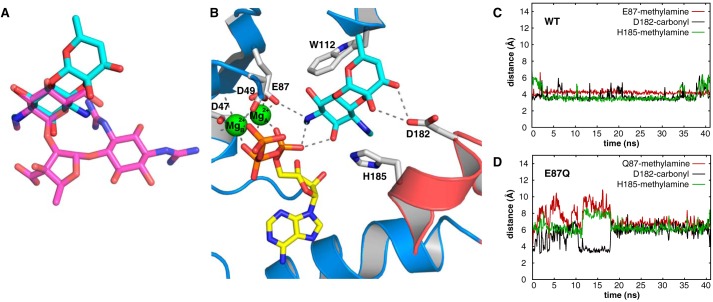
**Modeling of spectinomycin in the active site of AadA.** *A*, overlay of ring A of spectinomycin (*cyan*) onto ring C of streptomycin (*magenta*). *B*, average structure of AadA in complex with ATP, magnesium, and spectinomycin after MD simulation. Direct hydrogen bonds between protein and ligands are shown as *dashed lines. C* and *D*, distance plot of main interactions between AadA and spectinomycin along the MD simulations with the WT (*C*) or E87Q (*D*).

In previous ITC binding experiments, the AadA(E87Q) mutant did not show detectable binding of spectinomycin (12), and extensive soaking trials of WT AadA–ATP crystals with spectinomycin failed to produce interpretable electron density for the substrate. Therefore, spectinomycin was modeled in the active site of AadA based on the assumption that the modified hydroxyl groups of both substrates (Fig. 1) would be similarly positioned in the active site. In this model, the A ring of spectinomycin would overlay with the C ring of streptomycin with its 9-hydroxyl and one of the methylamines in equivalent positions as the 3″-hydroxyl and the methylamine from streptomycin (Fig. 7*A*). This ring would be positioned between Trp-112 and His-185 and held in place by a network of water molecules and the catalytic Glu-87 that interact with the 3″-hydroxyl and the methylamine group (Fig. 3*B*). WT and E87Q versions of the docked structure were subjected to 40-ns MD simulations using periodic boundary conditions and the OPLS-AA force field (24).

The proposed binding mode for spectinomycin was stable along the simulations for the WT version of the enzyme with an r.m.s.d. of 2.4 Å as compared with the initial docking pose. Indeed, after minor readjustments where spectinomycin moved 2 Å further into the binding site, the ligand remained anchored through hydrogen-bond interactions between Glu-87 and the methylamine group of ring A and Asp-182 and the carbonyl and hydroxyl groups of ring C. Additionally, the A ring of spectinomycin is stabilized by a π–cation interaction between His-185 and the positively charged methylamine group in position 6 and a hydrophobic interaction of Trp-112 at the opposite side of the ring (Fig. 7, *B* and *C*). In this position, the hidden van der Waals area of spectinomycin is 295 Å^2^ (59%). Conversely, in simulations of the complex with AadA(E87Q), ligand binding was unstable (r.m.s.d. of 3.6 Å), and after less than 10 ns the main interactions described above for the WT enzyme were lost (Fig. 7*D*), in agreement with experimental ITC data (12).

Spectinomycin in this binding mode packed against the N-terminal domain of AadA, far away from Trp-173 and Asp-178 and with only Asp-182 from the C-terminal domain at hydrogen-bond distance from the hydroxyl and carbonyl groups of its C ring. This could explain the conservation of Asp-182 and the neighboring Glu-183 (Fig. 7*A*) that through hydrogen bonds with backbone amides 181 and 183 stabilize the loop conformation that positions Asp-182. The importance of the suggested interactions is further supported by previous ITC data showing that the D182N mutation increased the *K_d_* for spectinomycin from 2 μm for WT AadA to >30 μm but showed no effect on affinity to streptomycin (12). However, after MD, the position of the target 9-hydroxyl is still at 5.1 Å from the α-phosphorus of ATP, and some further conformational change will be required before catalysis.

### Concluding remarks

This study exemplifies how binding specificity toward two substrates with limited similarity has evolved within the same active site and using partly the same residues. For both spectinomycin and streptomycin, the modified 3″- and 9-hydroxyl groups as well as the chemical groups to which AadA form direct interactions are also involved in direct hydrogen bonds with their respective binding sites in the 30S ribosomal subunit (4, 5), making it challenging to develop variants of these drugs that would retain binding to the ribosome but not be recognized by the ANT(3″)(9) enzymes. Based on detailed structural information on antibiotic-modifying enzymes, it would still be possible to explore differences in the steric boundaries of the rRNA- and protein-binding sites to expand the molecules in directions where activity is retained but binding to resistance-mediating enzymes is blocked.

## Experimental procedures

### Protein expression and purification

WT and mutant AadAs with C-terminal hexahistidine tags were expressed and purified as described previously (12).

### Crystallization and structure determination

All crystallization was done at 8 °C using the sitting-drop vapor-diffusion method. For the WT structures, 13.4 mg/ml WT AadA in 5 mm ATP and 10 mm MgCl_2_ was crystallized with a reservoir solution containing 10% (w/v) PEG 20,000, 20% (v/v) PEG monomethyl ether 550, 0.1 m MOPS/HEPES-Na, pH 7.5, or 0.1 m Bicine/Trizma (Tris base), pH 8.5, and 0.02 m each sodium l-glutamate, dl-alanine, glycine, dl-lysine HCl, and dl-serine (Morpheus screen, Molecular Dimensions). Crystals were vitrified in liquid nitrogen directly from the drop or after a 2-min soak with 5 mm streptomycin added to the drop. For the AadA(E87Q)–antibiotic complexes, 4.0–5.3 mg/ml protein in 5 mm ATP, 10 mm MgCl_2_, and 5 mm streptomycin or dihydrostreptomycin (Sigma-Aldrich) was crystallized with a reservoir solution containing 20% (w/v) PEG 6000, 0.2 m CaCl_2_, and 0.1 m HEPES, pH 7.5. Crystals of AadA(E87Q) with streptomycin were cryoprotected in 10% (w/v) PEG 20 000, 20% (v/v) PEG monomethyl ether 550, 30 mm MgCl_2_, 30 mm CaCl_2_, and 0.1 m Bicine/Trizma, pH 8.5, and crystals with dihydrostreptomycin were cryoprotected in reservoir solution supplemented with 10% glycerol, both containing 5 mm antibiotic before vitrification in liquid nitrogen.

Initial diffraction data were collected at the Diamond Light Source, Didcot, UK. Diffraction data for the deposited structures were collected at the European Synchrotron Radiation Facility, Grenoble, France. Data were processed with XDS (25) and scaled with AIMLESS (26). The structures were solved by molecular replacement with Phaser (27), the AadA–ATP structure with Protein Data Bank (PDB) code 5g4a (12) as search model and the antibiotic complexes with AadA–ATP as search model. All structures had two molecules in the asymmetric unit. The structures were refined by reciprocal-space refinement in Phenix (28) and model building and real-space refinement in Coot (29). Statistics for data processing and refinement are shown in Table 2. The structure factors and refined coordinates have been deposited in the PDB.

**Table 2:** **Data collection and refinement statistics**

	AadA–ATP	AadA(E87Q)–ATP–dhs	AadA(E87Q)–ATP–sry*^a^*	AadA–ATP–sry
**Data collection*^b^***				
Beamline	ID29	ID23-2	ID23-2	ID29
Wavelength	0.972	0.873	0.873	1.074
Space group	P3_2_	P3_2_	P3_2_	P3_2_
Unit cell parameters				
*a*, *b*, *c* (Å)	82.3, 82.3, 79.1	82.9, 82.9, 79.8	82.5, 82.5, 79.2	82.7, 82.7, 79.9
α, β, γ (°)	90, 90, 120	90, 90, 120	90, 90, 120	90, 90, 120
Resolution (Å)*^c^*	36.52–1.90 (2.00–1.90)	36.78–1.40 (1.50–1.40)	36.58–1.73 (1.83–1.73)	41.33–2.05 (2.10–2.05)
*R*_meas_ (%)*^c^*	15.4 (90.3)	8.2 (75.7)	17.1 (92.8)	11.2 (178.4)
〈*I*/σ(*I*)〉*^c^*	13.6 (2.52)	11.4 (2.14)	5.22 (0.98)	13.43 (1.33)
CC1/2 (%)*^c,d^*	99.8 (90.3)	99.8 (72.7)	98.9 (27.3)	99.9 (55.2)
Completeness (%)*^c^*	96.9 (90.2)	100 (100)	97.0 (99.2)	100 (99.9)
Redundancy*^c^*	9.7 (9.1)	5.2 (5.2)	2.6 (2.6)	10.3 (10.6)

**Refinement**				
Resolution (Å)	36.52–1.9	36.78–1.40	36.57–1.73	41.34–2.05
Reflections/test set	46,747/2,337	120,743/5,960	121,007/6,130	38,245/1,909
*R*_work_/*R*_free_ (%)	17.6/22.2	14.6/17.8	18.9/22.6	17.4/20.8
No. atoms	4,925	5,377	5,039	4,544
Protein	4,282	4,499	4,349	4,177
Ligand/ion	113	166	168	174
Water	530	712	522	193
B-factors				
Protein	31.2	21.6	29.2	50.1
Ligands	32.8	19.5	29.8	60.7
Solvent	38.6	31.1	35.3	45.2
r.m.s.d. from ideal				
Bond lengths (Å)	0.007	0.014	0.007	0.013
Bond angles (°)	0.907	1.381	0.892	0.660
Ramachandran plot				
Preferred (%)	99.2	98.6	98.8	98.5
Allowed (%)	0.6	1.38	1.2	1.5
Outliers (%)	0.2	0	0	0

**PDB code**	5g4a	5lpa	5luh	6fzb

*^a^* This structure was refined against data with separate Friedel pairs.

*^b^* Each data set was collected from a single crystal.

*^c^* Highest-resolution shell is shown in parentheses.

*^d^* CC1/2: correlation coefficient between intensity estimates from half datasets.

### Structure analysis

Detailed structure comparisons were done using secondary structure matching (SSM) in Coot (29) and the least squares (LSQ) commands in O (30). Structure figures were made using PyMOL version 1.7 (Schrödinger, LLC). Conformational changes were analyzed with DynDom (31). van der Waals contacts were quantified with AREAIMOL (32).

### Construction of chromosomal aadA mutations in S. enterica

The two mutations, W173A and D178A, were constructed using DIRex (33) using the primer and template combinations indicated in Table S1 to generate two PCR fragments for each mutation. The two overlapping PCR fragments (fragments 1 and 2 in Table S1) were coelectroporated in equimolar amounts into λ Red-induced DA27238 (WT *S. enterica* serovar Typhimurium strain LT2 containing the recombineering plasmid pSIM5-*tet* (34)) to generate a semistable DIRex intermediate containing an *AcatsacA* cassette (*cat-sacB*, conferring resistance to chloramphenicol and sensitivity to sucrose, flanked by inverted copies of the gene encoding the blue chromoprotein AmilCP) between directly repeated copies of the 25-bp sequence just next to the mutant position. Blue chloramphenicol-resistant transformants were streaked on sucrose selection plates (lysogeny broth agar without NaCl supplemented with 50 g/liter sucrose) to isolate white sucrose-resistant segregants that had lost the *AcatsacA* cassette. Segregants were verified by local sequencing of *aadA*.

### MIC determinations

As *aadA* is normally only expressed in minimal medium (34), MIC determinations were performed with cultures grown in liquid M9 medium with 0.2% (w/v) glycerol as carbon source (M9 + glycerol). Overnight cultures were grown in M9 + glycerol at 37 °C, diluted 500-fold into the same medium, and swabbed onto M9 + glycerol plates. Etest strips were applied to the plates, and the MICs were determined as the lowest concentration where no growth was visible after 16 h of incubation at 37 °C. The values in Table 1 are the medians of three independent cultures of each strain.

### Site-directed mutagenesis

AadA mutants W173A and D178A were generated by site-directed mutagenesis of pEXP5-CT-*aadA* (12) using the QuikChange II protocol (Stratagene) using the primers in Table S1. Mutations were confirmed by DNA sequencing.

### Thermofluor assays

The protocol was adapted from Niesen *et al*. (35). Each 25-μl reaction consisted of 10 μm WT or mutant AadA, 1 mm ATP, 5 mm MgCl_2_, 50 mm Tris-HCl, pH 7.5, 200 mm NaCl, 5 mm β-mercaptoethanol, 0.2 μl 50× SYPRO Orange dye, and 0–2000 μm streptomycin or spectinomycin. Reactions were done in triplicate in a Bio-Rad CFX Connect real-time system and subjected to a temperature gradient from 15 to 95 °C with an increment of 1 °C/30 s. Data analysis was done in CFX Manager software. The maximum difference between individual *T_m_* values and the mean *T_m_* was 0.7 °C.

### Molecular dynamics simulations

All-atom MD under explicit solvent were performed with the NAMD package (v.2.7) (36) using the OPLS-AA force field (24) and the TIP3P water model (37). The parameters for the OPLS-AA ligand were obtained from a minimization with Macromodel (Schrödinger LLC, 2009) and translated into the NAMD syntax with *ad hoc* scripts. Preparation of the systems and all MD analyses were conducted with the VMD package (38) and associated plugins. Each system was solvated in a cubic box that extended at least 12 Å away from any solute atom and neutralized with a 0.01 m concentration of NaCl. This allowed the use of periodic boundary conditions in combination with the particle-mesh Ewald algorithm to account for the long-range electrostatic interactions beyond a cutoff of 10 Å. A smoothing switching function was defined at 8 Å to smooth the boundary effects of this cutoff for all nonbonded interactions. The equilibration phase consisted of (i) energy minimization of the system (1000 steps), (ii) MD (25 ps long) in the NVT (canonical) ensemble where the temperature was gradually increased from 150 to 300 K, (iii) MD (25 ps long) in the NPT (isothermal-isobaric) ensemble using the Langevin piston method with a damping coefficient of 0.1 ps^−1^ for the conservation of the pressure (at 1 atm target value) and the temperature (at the target value of 310 K). The production phase followed for 40 ns with the same parameters as described for the last equilibration stage except for the time step, which was increased from the initial value of 1 fs to 2 fs, in combination with the SHAKE algorithm used to constrain bond lengths.

### Sequence analysis

Multiple sequence alignment was done using Clustal Omega (39) and manually modified with Jalview (40) to eliminate gaps in regions of defined secondary structure. Fig. 5 was prepared using the ESPript server (41).

### Malachite green assay

Reaction mixtures consisted of 50 mm Tris-HCl, pH 7.5, 200 mm NaCl, 5 mm MgCl_2_, 0.5 mm tris(2-carboxyethyl)phosphine, 0.2 mm streptomycin, 0.2 mm ATP, 0.3 unit/ml pyrophosphorylase (Sigma-Aldrich), and variable concentrations of AadA. The reaction was initiated with enzyme in a total reaction volume of 190 μl and incubated for 15 and 35 min followed by mixing 60 μl of reaction mixture with 15 μl of malachite green solution (P_i_ ColorLock^TM^ Gold Mix, Innova Biosciences) and after 5 min with 5 μl of stabilizer (P_i_ ColorLock Stabilizer, Innova Biosciences). Malachite green complex absorbance at 620 nm was measured using an EnVision spectrophotometer (2104 Multilabel reader, PerkinElmer Life Sciences) in 96-well plates. For the final assay, protein concentration and time were adjusted to reach linear values suitable for specific activity calculations. WT enzyme concentrations used were 0.25, 0.5, and 1 μm. E87A mutant concentrations were 1, 2, and 4 μm. E87Q mutant concentrations were 0.25, 0.5, and 1 μm. Specific activity was calculated using a phosphate calibration curve ranging from 2 to 40 μm.

### Chromatographic adenylation assay

Reaction mixtures with WT enzyme or E87Q were incubated for 1 day with 2 mm streptomycin and 10 mm ATP in a buffer containing 50 mm HEPES, pH 7.5, 160 mm NaCl, 10 mm MgCl_2_, 1 mm DTT, and pyrophosphatase at room temperature with gentle agitation. Fresh AadA and pyrophosphatase were added at four time points during the incubation to end up with a final concentration of 0.03 mg/ml and 1.5 units/ml, respectively. After incubation, the enzymes were removed from the reaction mixtures by filtration with a Vivaspin 20 concentrator (polyethersulfone membrane, 10,000-Da molecular weight cutoff, Sartorius), and the antibiotic was washed out from the enzyme by successive dilutions in buffer A (20 mm MES, pH 6.0). 5 ml of filtrate was applied to a 1-ml HiTrap SP Sepharose Fast Flow column (GE Healthcare). Nonreacted ATP and AMP eluted upon wash with buffer A. Adenylated streptomycin was eluted with a 0–25% gradient of buffer B (20 mm MES, pH 6.0, 1 m NaCl) in 20 column volumes, and absorbance was monitored at 260 nm.

## Author contributions

A. L. S. and M. S. supervision; A. L. S., S. E. V. d. V., S. K. P., J. N., H. G. d. T, and M. S. investigation; A. L. S., H. G. d. T., and M. S. writing-original draft; M. S. conceptualization; M. S. resources; M. S. data curation; M. S. project administration; M. S. writing-review and editing.

## Supplementary Material

Supporting Information

## Footnotes

4 The abbreviations used are:

dhs: dihydrostreptomycin
ANT: aminoglycoside nucleotidyltransferase
ITC: isothermal titration calorimetry
MD: molecular dynamics
r.m.s.d.: root mean square deviation
AMPCPP: adenosine 5′-(α,β-methylene)triphosphate
AMPNPP: adenosine 5′-[(α,β)-imido]triphosphate
sry: streptomycin
MIC: minimum inhibitory concentration
Bicine: *N*,*N*-bis(2-hydroxyethyl)glycine
OPLS-AA: all-atom optimized potentials for liquid simulations
PDB: Protein Data Bank.
