# Supplementary material for: Structural mechanism of AadA, a dual-specificity aminoglycoside adenylyltransferase from Salmonella enterica
Source: J Biol Chem. 2018 Jun 5;293(29):11481–90. doi: 10.1074/jbc.RA118.003989 (PMC6065190; doi:10.1074/jbc.RA118.003989)
Supplement: Supporting Information [file supp_293_29_11481__index.html]

Structural mechanism of AadA, a dual specificity aminoglycoside adenyl transferase from Salmonella enterica — Substrate recognition of AadA — Structural mechanism of AadA, a dual-specificity aminoglycoside adenylyltransferase from Salmonella enterica — Substrate recognition of AadA — Supporting Information 

# Structural mechanism of AadA, a dual-specificity aminoglycoside adenylyltransferase from *Salmonella enterica*

## Supporting Information

- Supplemental material - Supplemental table S1, supplemental figures S1-S3
