# Supplementary material for: Structural mechanism of AadA, a dual-specificity aminoglycoside adenylyltransferase from Salmonella enterica
Source: J Biol Chem. 2018 Jun 5;293(29):11481–90. doi: 10.1074/jbc.RA118.003989 (PMC6065190; doi:10.1074/jbc.RA118.003989)
Supplement: Supporting Information [file supp_RA118.003989_138177_1_supp_147517_p9n5s6.docx]

**Supplemental material for**

**Structural mechanism of AadA, a dual specificity aminoglycoside adenyl transferase**

Ana Laura Stern^1#^, Sander Egbert Van der Verren^1,3#^, Sandesh Kanchugal P^1#^, Joakim Näsvall^2^, Hugo Gutierrez de Teran^1^ & Maria Selmer^1*^

^1^Department of Cell and Molecular Biology, Uppsala University, BMC, Box 596, SE-751 24 Uppsala, Sweden

^2^Department of Medical Biochemistry and Microbiology, Uppsala University, BMC, Box 582, SE-751 23 Uppsala, Sweden

^3^ Current affiliations: Structural and Molecular Microbiology, VIB-VUB Center for Structural Biology, Pleinlaan 2, 1050 Brussels, Belgium & Structural Biology Brussels, Vrije Universiteit Brussel, Pleinlaan 2, 1050 Brussels, Belgium
^#^ These authors contributed equally to this work

* to whom correspondence should be addressed: [maria.selmer@icm.uu.se](mailto:maria.selmer@icm.uu.se)

Contains supplemental table S1 and supplemental figures S1-S3

**Table S1. DNA oligos used in generation of mutations in the *aadA* gene**

| Oligo name | Sequence (5’ – 3’)^a^ | Comment |
| --- | --- | --- |
| *aadA*_W173A-fP1 | GGCCTTGCGTTATCCCCTCGACCTTctGCAATCCACGGCAGATGTGCAGGGA**GTGTAGGCTGGAGCTGCTTC** | Used with cat_midF to amplify *'cat-sacB-amilCP* from *Acatsac3*, fragment 1 for generating W173A |
| *aadA*_173-rP1 | GATATGATACTCATCTCCCTGCACATCTGCCGTGGATTGC**GTGTAGGCTGGAGCTGCTTC** | Used with *cat*_midR2 to amplify *cat'-amilCP* from *Acatsac1*, fragment 2 for generating W173A |
| *aadA*_D178A-fP1 | CTCGACCTTTGGCAATCCACGGCAGcTGTGCAGGGAGATGAGTATCATATC**GTGTAGGCTGGAGCTGCTTC** | Used with *cat*_midF to amplify *'cat-sacB-amilCP* from *Acatsac3*, fragment 1 for generating D178A |
| *aadA*_178-rP1 | CGCCAGGGTTAAAACGATATGATACTCATCTCCCTGCACA**GTGTAGGCTGGAGCTGCTTC** | Used with *cat*_midR2 to amplify *cat'-amilCP* from *Acatsac1*, fragment 2 for generating D178A |
| *cat*_midR2 | GCCGACATGGAAGCCATCAC |  |
| *cat*_midF | CGACGATTTCCGGCAGTTTC |  |
| *aadA*verF | GCCGTTATGCCATATTTCTG | Used with *aadA*verR for PCR and sequencing of *aadA* |
| *aadA*verR | ACTTCAGCGATGAGAACCTA | Used with *aadA*verF for PCR and sequencing of *aadA* |
| W173A_forward | TTGCGTTATCCCCTCGACCTTGCACAATCCACGGCAGAT | Used for QuickChange mutagenesis of pEXP5-CT-*aadA* |
| W173A_reverse | ATCTGCCGTGGATTGTGCAAGGTCGAGGGGATAACGCAA | Used for QuickChange mutagenesis of pEXP5-CT-*aadA* |
| D178A_forward | CTTTGGCAATCCACGGCAGCCGTGCAGGGAGATGAGTAT | Used for QuickChange mutagenesis of pEXP5-CT-*aadA* |
| D178A_reverse | ATACTCATCTCCCTGCACGGCTGCCGTGGATTGCCAAAG | Used for QuickChange mutagenesis of pEXP5-CT-*aadA* |
| ^a^ lower case letters indicate mutations, bold face letters indicate the part that acts as primer for PCR amplification. | | |


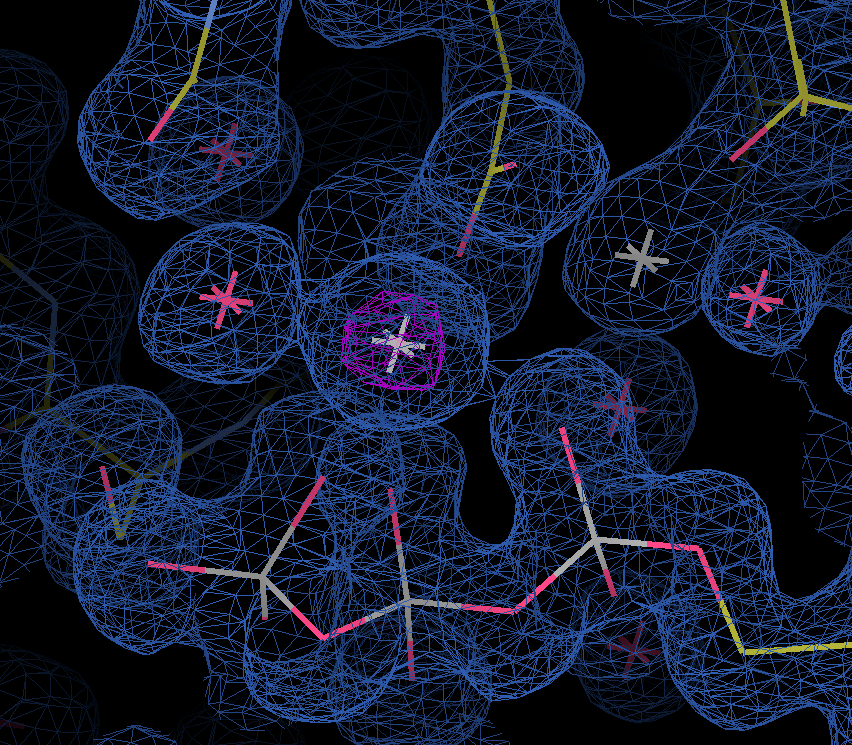


**Figure S1.** Anomalous difference map (magenta) countoured at 5 sigma shows clear signal for Ca_B_ (left) in the AadA(E87Q)-ATP-dhs structure.

**Figure S2.** Water coordination in the active site of *Sa*AadA E87Q with calcium, ATP and dihydrostreptomycin (dhs).

**Figure S3**. Coordination of divalent metal ions in the active site of *Sa*AadA in complex with ATP and magnesium (A, D) and *Sa*AadA E87Q in complex with ATP, calcium and dihydrostreptomycin (B, E) and *Sa*AadA in complex with ATP, magnesium and streptomycin (C, F).
